# HPV16 Phylogenetic Variants in Anogenital and Head and Neck Cancers: State of the Art and Perspectives

**DOI:** 10.3390/v16060904

**Published:** 2024-06-03

**Authors:** Luisa Galati, Paola Di Bonito, Mariarosaria Marinaro, Maria Vincenza Chiantore, Tarik Gheit

**Affiliations:** 1 International Agency for Research on Cancer, 69007 Lyon, France; 2 Department of Infectious Diseases, Istituto Superiore di Sanità, Viale Regina Elena 299, 00161 Rome, Italy; paola.dibonito@iss.it (P.D.B.); mariarosaria.marinaro@iss.it (M.M.); mariavincenza.chiantore@iss.it (M.V.C.)

**Keywords:** HNSCC, OPC, cervical cancer, HPV16, HPV variants, phylogeny, NGS

## Abstract

HPV16 is responsible for approximately 60% and 90% of global HPV–induced cervical and oropharyngeal cancers, respectively. HPV16 intratype variants have been identified by HPV genome sequencing and classified into four phylogenetic lineages (A–D). Our understanding of HPV16 variants mostly derives from epidemiological studies on cervical cancer (CC) in which HPV16 B, C, and D lineages (previously named “non-European” variants) were mainly associated with high-grade cervical lesions and cancer. Although a predominance of HPV16 lineage A (previously named “European variants”) has been observed in head and neck squamous cell carcinoma (HNSCC), epidemiological and in vitro biological studies are still limited for this tumor site. Next Generation Sequencing (NGS) of the entire HPV genome has deepened our knowledge of the prevalence and distribution of HPV variants in CC and HNSCC. Research on cervical cancer has shown that certain HPV16 sublineages, such as D2, D3, A3, and A4, are associated with an increased risk of cervical cancer, and sublineages A4, D2, and D3 are linked to a higher risk of developing adenocarcinomas. Additionally, lineage C and sublineages D2 or D3 of HPV16 show an elevated risk of developing premalignant cervical lesions. However, it is still crucial to conduct large-scale studies on HPV16 variants in different HPV–related tumor sites to deeply evaluate their association with disease development and outcomes. This review discusses the current knowledge and updates on HPV16 phylogenetic variants distribution in HPV–driven anogenital and head and neck cancers.

## 1. Introduction

Human papillomaviruses (HPVs) are non-enveloped DNA viruses that infect both cutaneous and mucosal epithelia. To date, over 400 papillomaviruses (PVs) have been identified and over 200 are HPV genotypes classified into alpha, beta, gamma, mu, and nu genera [1] (www.hpvcenter.se) (accessed on 13 March 2024). A clear association with human cancers has been established for the following mucosal alphapapillomavirus HPV types, namely HPV51, grouped in the alpha-5 species; HPV 56, in the alpha-6; HPV18, 39, 45, and 59, in the alpha-7; and HPV16, 31, 35, 33, 52, and 58, grouped as alpha-9 species. Therefore, they have been classified by the International Agency for Research on Cancer (IARC) monograph as carcinogenic (Group 1) or high risk (HR) HPV genotypes [2]. Among them, approximately 60% of cervical cancer and 90% of oropharyngeal HPV–driven cancers are attributable to HPV16.

**Figure 1:**
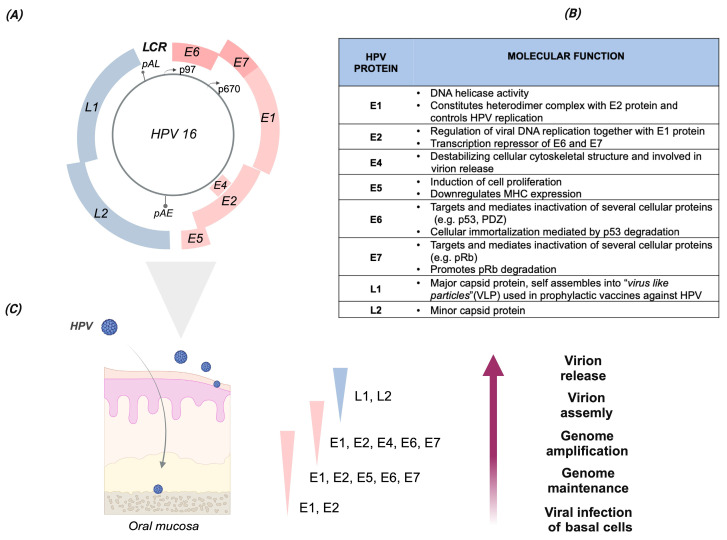
HPV16 genome organization, protein functions, and viral life cycle during productive infection. (**A**) Genome organization of the HPV16 genotype. The HPV genome is a double-stranded DNA (indicated by the gray circle), with a size of about 7900 bp. The major six early (E) open reading frames (ORFs), namely E6, E7, E1, E2, E4, and E5, and the two late (L) ORFs, namely L1 and L2, are indicated by different colors, with E6 and E7 shown in dark pink. Furthermore, the HR HPVs express an additional early protein, E8ˆE2C, by spliced mRNA. The major early p97 and late p670 promoters are indicated by arrows. The early and late polyadenylation sites, pAE and pAL, respectively, are also indicated by grey bar lines. The long control region, LCR (alternatively named the upstream regulatory region, or URR), comprises the replication origin and sequences involved in transcription. (**B**) HPV16 proteins. List of the HPV16 proteins and their principal functions. (**C**) HPV life cycle during productive infection. The viral life cycle during productive HPV infection in the host epithelial tissue (schematically represented on the left) is characterized by a specific pattern of viral gene expression across the epithelial layers. The viral life cycle is strictly regulated and linked to the host cell epithelial differentiation process. As reported for the cervical epithelium, HPV gains access to the basal layer through the epithelial transition zones (TZ) of the uterine cervix, in the presence of microlesions, wounds, or cuts. After the infection of basal cells, the HPV genome is maintained in the nucleus in an episomal state at a relatively low copy number. The expression of E6 and E7, through the p97 promoter, is necessary to start the viral life cycle. As the infected cells migrate to the upper epithelial layers, the viral proteins E1, E2, E4, and E5 are upregulated via the p670 promoter to facilitate viral genome amplification. In the upper epithelial layers, the expression of the late viral capsid proteins, L1 and L2, promotes capsid assembly and subsequent release of the new virion from the epithelial surface. Text and figures are based on the following manuscript: [15,16,17,18,19]. Created with BioRender.com.

In particular, the HPV16 genotype belongs to the Alphapapillomavirus genus, and it is included in species 9. Its circular double-stranded DNA genome comprises the following regions: (i) the long control region (LCR), containing genetic elements involved in viral replication and transcription; (ii) the early (E) region encoding for the non-structural proteins E1, E2, E4, E5, E6, and E7 that are involved in fundamental viral processes, with E6 and E7 responsible for HPV oncogenicity; and (iii) the late (L) region, encoding the structural proteins L1 and L2, which are, respectively, the major and minor viral capsid proteins (Figure 1A–C).

The classification of HPV into genera, species, and genotypes is primarily based on the nucleotide sequence of the L1 ORF, the most conserved gene, which encodes the major viral capsid protein [3,4]. A minimum of 60% identity in the L1 nucleotide (nt) sequence defines HPV types belonging to the same genus. Viruses with L1 nt sequence identity between 71% and 89% belong to the same species (e.g., *Alphapapillomavirus* species 9), while viruses showing more than 90% L1 nt identity are defined as distinct genotypes (e.g., HPV16 or HPV18).

**Figure 2:**
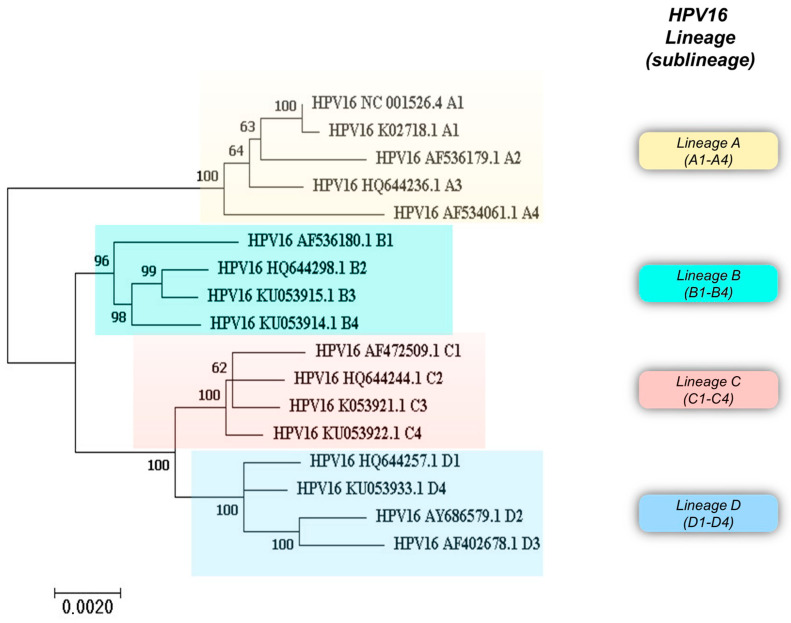
Phylogenetic analysis of HPV16 variant reference sequences with the corresponding GenBank accession number. Phylogenetic analysis of HPV16 variant sequences was performed with MEGA 7.0.26 software. The tree was built using the Maximum Likelihood method.

Additionally, HPV intra-genotype variants have been identified by sequencing and phylogenetic analysis of the entire viral genome and classified into lineages when the genome variability ranges from 1% to 10% (HPV16 lineages A–D), and into sublineages when the variability ranges from 0.5% to 1% (HPV16 sublineages A1–4, B1–4, C1–4, and D1–4) [3,5] (pave.niaid.nih.gov accessed on 13 March 2024). HPV16 variant classification and their phylogenetic stratification into lineages and sublineages are reported in Table 1 and Figure 2.

Studies on the role of HPV16 variants were mainly focused on cervical cancer, highlighting a link between specific phylogenetic HPV variants and a higher cancer risk. Some HPV16 lineages/sublineages were reported to be preferentially associated with an increased risk of cancer, such as HPV16 sublineages D2, D3, or A4. They are also found to be associated with an increased risk of adenocarcinoma. Moreover, the role of single nucleotide polymorphisms (SNPs) in the HPV16 genome, such as the E6 T350G that leads to the amino acid substitution L83V, has been investigated to understand their role in cervical disease progression and viral persistence. Thus, investigating HPV genome variability could be beneficial to further exploring the possible association with HPV–related tumors and/or with the disease outcome.

HPV does not encode its own DNA polymerase. Instead, it recruits high-fidelity host enzymes for viral genome synthesis, resulting in a low mutation rate across the HPV genome. DNA mutations occur differently in coding and non-coding viral genomic regions. In coding regions, the estimated mutation rate ranges from 2 × 10^−8^ to 5 × 10^−9^ substitutions per site/year, whereas in non-coding regions, the rate is twice as fast, as reviewed in [6].

Moreover, during viral infection, as a part of the innate immune response of the host, the HPV genome can be targeted by cytosine deaminases of the apolipoprotein B mRNA editing catalytic polypeptide-like 3 (APOBEC3 or A3) family, which includes A3A, A3C, A3H, A3B, A3D, A3F, and A3G [7,8]. The APOBEC A3 enzymes catalyze the cytosine-to-uracil conversion, leading to a thymidine substitution during the viral replication. They act as viral restriction factors to clear the infection. Human APOBEC3 enzymes can inhibit a broad spectrum of viruses, such as HIV, HBV, HHV-1, and HHV-4 [7,9,10,11]. Some studies have reported that a group of viruses, including HR HPV types, have evolved mechanisms to induce the upregulation of some APOBEC3 family members [11,12,13]. Thus, HPV variants may arise due to mutations driven by the APOBEC activity, which targets the viral genome during the infection and may accidentally contribute to viral evolution. Alternatively, HPV variants may be selected and evolve to evade APOBEC activity by reducing APOBEC3 target sequences in their genome [14].

Here, the role of HPV16 phylogenetic variants in HPV–related cancers is reviewed and the implications of variants in anogenital and head and neck cancers are also discussed based on recent NGS findings.

## 2. Methodology

The aim of the present review was to give an overview of the most investigated HPV16 SNPs, variant lineages, and sublineages and their geographic distribution, and to describe their possible link with HPV–related cancers. Moreover, the comprehensive review presented here will be helpful in exploring the role of HPV16 phylogenetic variants and their contribution to cancer progression. The literature search strategy relied only on the use of PubMed with the following keywords: “HPV16 variants”, “HPV16 lineages”, “HPV16 sublineages”, “HPV16 SNPs”, “HPV16 variants and cervical cancer, “HPV16 variants and head and neck cancer”, “HPV16 variants and oropharyngeal cancer”, “HPV16 variants and HPV-associated cancers”, and “HPV16 variants and next generation sequencing”. In the present manuscript, peer-reviewed reviews, meta-analyses, and original research articles were included. Non-peer-reviewed sources, articles not available in English, and studies not including the HPV16 genotype were not included in this review.

## 3. HPV16 Variants, Lineages, and Their Classification

Overall, analysis of the whole genome sequence of HPV16 has revealed characteristic mutations that categorize HPV16 isolates into four phylogenetic lineages, namely A, B, C, and D, subdivided into 16 sublineages: A1 to A4, B1 to B4, C1 to C4, and D1 to D4. Figure 2 shows a phylogenetic tree built using HPV16 variant reference sequences retrieved from the Pave database (https://pave.niaid.nih.gov) (accessed on 13 March 2024) [5].

The HPV16 variants have been geographically classified depending on the place of their initial identification; in fact, they were named European (E), Asian American (AA), African-1, and African-2 (Af-1 and Af-2) variants [20]. According to this first classification, the European lineages appear to be the most prevalent worldwide [21], showing a large diffusion in Europe, North and South America (sublineages A1–3), and Asia (sublineage A4), and with a minor representation in Africa, where B and C variants predominate [22,23]. Recently, the novel A5 subvariant was identified in cervical samples from Japanese women [24], although its corresponding sequence has yet to be reported in the Pave database (https://pave.niaid.nih.gov) (accessed on 13 March 2024).

An alphanumeric system is now used to classify HPV16 variants [22], while geographic classification is no longer recommended, as reported by Burk et al. and Mirabello et al. [5,25] (Table 1). Indeed, to avoid misleading geographic nomenclatures of HPV variants and facilitate cross-study comparisons, adherence to the new classification based on lineages (A up to D) and sublineages (A1–up to D–4) is recommended [25].

Additionally, Cornet et al. (2012) [23] have reported a combination of SNPs located in the E6 and LCR regions specific to each of the HPV16 sublineages, which they proposed as “diagnostic” SNPs. Yet, some non-lineage-specific SNPs were also found in the HPV16 sublineages, such as the most studied T350G nt variation [23]. Moreover, a larger spectrum of SNPs occurring in the viral early genes, namely E1, E2, E4, E5, E6, and E7, and in the LCR was reviewed by Bletsa et al. (2021) [26]. Focusing on E6, a pattern of SNPs in this viral ORF was found in each of the HPV16 sublineages, such as the A1 and A2 sublineages (T350G), the A4 sublineage (T178G), the B lineage (G132C, C143G, G145T, T286A, A289G, and C335T), the C lineage (T109C, G132T, C143G, G145T, T286A, A289G, C335T, and G403G), and the D lineage (G145T, T286A, A289G, C335T, T350G, and A532G) reviewed in [26]. Also, the nt sequence analysis of E7 revealed specific nucleotide variations related to the following lineages: A4 sublineage (A647G), B lineage (T789C and T795G), C lineage (A647G, T789C, and T795G), and D lineage (T732C, T789C, and T795G), reviewed in [26]. Most of the SNPs located in the E6 and E7 genes are described in the following paragraphs and in Table 2. Mainly SNPs in HPV E6 and E7 ORFs have been investigated, as these genes encode for oncoproteins that facilitate the degradation of essential tumor suppressor cellular proteins, such as p53 and pRb, involved in the cell cycle, apoptosis, and cellular proliferation pathways [15] (Figure 1B). Therefore, E6 and E7 are recognized as the major viral oncoproteins, since they are the main drivers of HPV–mediated carcinogenicity.

## 4. HPV16 Variants and Cervical Cancer

HR HPV genotypes are associated with various anogenital cancers, i.e., cervical, vaginal, vulvar, penile, and anal cancers, as well as oropharyngeal squamous cell carcinoma (OPSCC). HR HPV genotypes display different oncogenicity, with HPV16 being the most prevalent type detected in premalignant and malignant lesions [2,41] and responsible for the majority of cervical cancers worldwide [42,43]. Epidemiological studies conducted in patients with cervical cancer have provided evidence that HPV16 variants differ in (i) geographical distribution, (ii) persistence in the infected host, and (iii) ability to favor progression to cancer [44,45,46].

In the next subsections, data from studies exploring HPV variant distribution and persistence in the host are discussed; the sturdiness and advancement of research studies are highlighted together with the connection between viral variants and disease outcome.

### 4.1. HPV Variant Distribution and Persistence in the Host

Focusing on geographical variant distribution, the HPV16 sublineages A1, A2, and A3 are the most prevalent worldwide and are responsible for the majority of HPV16 infections as observed by analyzing cancer samples and healthy controls [23,47,48]. The persistence of HPV16 infection in the host is a key step for viral-mediated carcinogenesis. Previous studies have often compared “European” to “non-European” HPV16 variants and found that non-European variants exhibit an increased risk of viral persistence [46,49,50,51,52]. As reported in a 3-year longitudinal follow-up study based on HPV16 whole-genome Sanger sequencing, infections with variants other than A1–A2 (the most common “European” sublineages) are preferentially cleared by the host. Moreover, single nucleotide polymorphisms (SNPs) across the HPV16 genome did not affect clearance or viral persistence, suggesting that the progression to tumor could be host-related [53]. The risk of HPV16 persistence in the infected host has been extensively studied, focusing on specific viral regions; e.g., E6 nucleotide polymorphism 350T versus 350G. As described above, this nucleotide mutation results in AA change from valine to a leucine (L83V) at position 83 in the E6 viral protein. Recently, the presence of the T350G variant has been associated with progression to high-grade lesions and with an elevated risk of developing CC in a study conducted in Argentina [54].

The prevalence of the E6 variation in HPV16 sublineages A1–A3 has shown a higher mutation rate of T350G in Central/South America compared to a European cohort, particularly in cervical and penile cancers [47]. In vitro biological studies supported the epidemiological findings [55,56,57,58,59]. However, it has become evident that the oncogenicity of HPV16 E6 variants could be population-dependent [60,61], underlining a role played by the host’s genetic background [60,62,63,64]. In addition, a two-fold increase in the risk of HPV persistence has been observed for the European E6 350T prototype in a European cohort [64,65].

Recently, E6 AA changes have been studied by using several machine-learning approaches to predict the development of high-grade cervical lesions (H-SIL) [66]. These in silico findings indicate that D32E and H85Y AA mutations in the E6 protein result in an increased ability to degrade p53 when compared to the E6 prototype [66].

### 4.2. Sturdiness and Advancement

To better discriminate the variability within the HPV genome sequence, large-scale studies based on NGS techniques have been designed [52,63,67,68]. This sequencing methodology allows for high throughput testing and facilitates full analysis of the entire HPV genome to unravel mutations.

Mirabello and collaborators assessed HPV16 variant lineages and their association with the risk of developing cervical precancer and cancer in 3200 enrolled women [63]. They showed that sublineage A4 was associated with an increased risk of developing adenocarcinoma, whereas lineage C showed an elevated risk of developing cervical premalignant lesion CIN3, as did the D2–D3 sublineages, which are also associated with an increased cancer risk compared to the A1–A2 sublineages [63]. Furthermore, the study showed an increased risk of developing precancerous or cancerous lesions when the ethnicity of the patients matched the geographical origin of the infecting HPV16 variants [63]. In another study conducted by Clifford et al. (2019), the HPV16 D2 and D3 sublineages, along with A3 and A4, showed an increased risk of cervical cancer compared to the A1 sublineage [48]. In a study conducted in Guatemala by Lou et al. (2020), the HPV16 D2 and D3 sublineages were frequently observed in cervical tumors and in adenocarcinoma histological type [69]. Moreover, in cancers harboring the HPV16 D2 sublineages, the authors reported a higher rate of viral DNA integration into the host genome [69].

In a study conducted in Japan by whole genome sequencing (WGS) on HPV16 isolates from cervical samples collected from women with and without cervical malignancies and invasive cervical cancer, Hirose et al. (2019) reported a prevalent clustering of HPV16 isolates mainly in sublineages A4 (52%) and A1 (21%) [24]. Sublineage A4 showed a significantly higher risk for cervical cancer development compared to other A sublineages [24]. In addition, the A4 sublineage was frequently detected in invasive CC (73.2% of cases), and a higher risk of progression from premalignant lesions (CIN2-3) to cervical squamous cell carcinoma was found for this sublineage compared to HPV16 clades A1, A2, and A3 [24].

### 4.3. Disease Outcome

Exploring the role of HPV variants in premalignant and malignant cervical lesions, HPV16 B, C, and D lineages (known as the “non-European” variants) have been shown to be prevalent in high-grade lesions and cervical squamous cell carcinoma [44,50,51,70,71]; however, some studies have reported a lack of association with cervical disease [72,73,74]. In a large study across Europe, Asia, and Central/South America, HPV16 sublineages A1–A3 were shown to be the most prevalent in cervical, vaginal, and penile cancers, regardless of the geographical origin of patients, while sublineage A4 was mainly associated with anal cancer in Asian cohorts [47]. Recently, in the context of the HPV Infection in Men (HIM) studies, a high prevalence of the HPV16 A1 sublineage was found in the anal swabs from men with anal cancer from Brazil, Mexico, and the United States [75], with no significant differences observed between variant lineages and HPV16 persistence [75].

The association between HPV16 sublineages and cancer histology showed an increased risk of developing an adenocarcinoma for the A4 sublineage, while the D2–D3 HPV16 sublineages were strongly associated with an increased risk of developing premalignant CIN3 lesions and cervical cancer, with the strongest risk of adenocarcinomas linked to the D2 sublineage [25,63]. The HPV16 D clade was also prevalent in adenocarcinomas [76,77] from South/Central and North American patients [48], although these findings were not confirmed by De Boer et al. when E6 and L1 were sequenced in a relatively small group of HPV16–positive adenocarcinoma samples [78].

Genomic characterization of 228 primary cervical cancers within “The Cancer Genome Atlas (TCGA)” program showed a predominance of European HPV16 variants (primarily the A1 variant), whereas non-European variants (sublineages A4, B1, C1, D2, and D3) were significantly associated with cervical adenocarcinomas [79].

## 5. HPV16 Variants and Head and Neck Tumor

Head and neck cancers rank as the sixth most common cancer type globally [80], with an increasing incidence of HPV–driven OPSCC in developed countries, particularly among men [81]. Alcohol, smoking, and persistent HR HPV infections are the major risk factors for HNSCC. A recent systematic review and meta-analysis has provided evidence that artificial intelligence (AI) using image-based analysis can be a promising tool for predicting HPV status in HNSCC [82]. However, its accuracy is still lower compared to the p16^INK4a^ immunohistochemistry, a reference diagnostic method in HPV-related OPC [82]. Among HR HPVs, the majority (up to 90%) of HPV–driven OPSCCs are related to HPV16 infection. The prevalence of HPV–induced OPSCCs varies geographically, ranging from 22 to more than 74% [83], whereas only 2.0–3.9% of oral and 2.0–3.1% of laryngeal cancers are attributed to HR HPV infections [83,84,85]. Although HPV16 variants have been extensively studied in cervical cancer, less is known about their significance in head and neck cancers [86]. A list of studies conducted on HNSCC and published in the last 23 years is reported in Table 2.

A study conducted in Greece showed that 85% of HPV16 sequences detected in 40 specimens from subjects with HNSCC were clustered into the European sublineage A3, while the remaining 15% of HPV16 variants were related to sublineage D1 [35]. In another study on HPV16–positive OPSCCs performed in the USA, the A1 sublineage was associated with poor recurrence-free survival (RFS) [87]. Conversely, variants other than the HPV16 A1 sublineage are correlated with improved RFS, particularly in moderate or low tobacco smokers [87].

A recent systematic review of studies on head and neck cancers and HPV16 variants using geographical nomenclature and including studies from the USA (n = 3), Germany (n = 1), Italy, (n = 2) Brazil (n = 1), Japan (n = 1), and Iran (n = 1) revealed a predominance of European variants in HNSCCs, followed by Asian American and African lineages [86]. Therefore, even though a predominance of European strains has been reported, no correlation with patient prognosis has been made [86].

In a comparative study conducted in the USA on HPV16 variant distribution in CC and OPSCC by grouping both European and Asian (E plus A) variants, the authors showed that this combined variant group was more prevalent in OPSCC compared to CC [32]. Conversely, the group of Asian American (AA1 plus AA2) HPV16 variants prevailed in cervical samples. In addition, non-synonymous mutations in the E6 protein showed significantly higher prevalence rates in OPSCCs, while E7 nucleotide sequences showed fewer mutations in both cancer types [32].

Conversely, an Italian study reported a prevalence of 19.6% for African HPV16 variants in HNSCC samples, highlighting their relevance in the head and neck anatomical tumor site [30]. As seen before in CC studies, when focusing on specific viral genome regions, such as the E6 gene, the most frequent polymorphism reported in a small cohort of HNSCCs was T350G [35,37]. However, this finding needs further investigation to determine its impact on clinical outcomes. Others have compared the frequency of the E6 polymorphisms in tonsillar squamous cell carcinoma (TSCC), CC, and cervical samples from Swedish patients [39], and reported that the R10G amino acid change (nt A131G) was frequent in TSCC, rare in cervical samples, and absent in CC, with no significant differences found in 3-year disease-free survivors among patients. In addition, European E6 variants carrying the L83V (nt change T350G) mutation were detected across all cancer types, with no significant correlation found with disease-free survivors among the patients [39].

Finally, a comparative study performed in the USA based on E6 sequencing data from oral rinse samples and matched tumor tissues showed that the most frequent variants were European [29], with the E6 E-350T prototype (n = 6) being the most prevalent in oral rinse samples, followed by the E6 variant E-350G (n = 4).

To date, very few NGS–based studies on HPV variants in HNSCC specimens have been conducted [33,34]. Thus, further studies are needed to elucidate the possible impact of HPV16 phylogenetic variants on HNSCC and particularly in OPC. In a large USA–based study focused on HPV16–positive OPSCC, whole-genome NGS sequencing identified A1 as the most prevalent sublineage, although no correlation between HPV variant lineages and histological subtypes was reported [33]. In a recent study performed in the USA on a cohort of OPC patients, the majority of the HPV16 variants belonged to the A lineage (90.3% of cases) [34]. Among them, A1 was the most common sublineage, being detected in more than a half of the cases, followed by A2 (27.8%), D3 (6.5%), and A4 (5.7%) [34]. The most important findings in this study include eight SNPs, observed in some HPV genes, significantly associated with reduced patient survival. These polymorphisms were found in the viral E1 gene (nt position 1053), with four in the L2 gene (nt positions 4410, 4539, 5050, and 5254), two in the L1 gene (nt position 5962 and 6025), and one in the LCR region (nt position 7173) [34]. The latter was strongly associated with an increased mortality hazard rate. These results indicate that nucleotide variations across the HPV16 genome can impact the prognosis of HPV–positive OPC patients [34]. However, HPV infection is a necessary but insufficient condition for cancer development, which is a multifactorial event involving lifestyle, environmental, and genetic host factors.

Moreover, in recent years, evidence suggests that mutations in certain host genomic loci (e.g., HLA) are associated with either cervical cancer or head and neck cancer susceptibility [88,89]. Nevertheless, the precise contribution of these mutations in cancer development remains unknown, and specific studies on this topic need to be addressed [90].

## 6. Conclusions

To date, the majority of studies aimed at exploring the significance and distribution of HPV variants have been designed and mainly conducted on cervical cancer. Overall, some limitations should be highlighted, including the small sample size analyzed in some studies, the paucity of current studies of HPV variants in HPV–associated cancers other than cervical cancer, the shortage of NGS–based investigations, together with the absence of mechanistic biological studies focusing on specific HPV16 SNPs.

The main findings from the cervical cancer studies could be summarized as follows: the HPV16 sublineages, namely D2, D3, A3, and A4, show an increased risk of cervical cancer development, with the HPV16 sublineages A4, D2, and D3 mainly associated with an increased risk of developing adenocarcinomas. Also, studying the HPV genome variability by NGS-WGS in head and neck cancer specimens has revealed associations between different HPV16 SNPs (e.g., E1, L1, L2, and LCR) and disease prognosis in HPV–related OPC. The potential impact of HPV variants on cancer development at different anatomical sites and their association with disease outcome remains largely unexplored and needs further investigation. Finally, both NGS techniques and the current alphanumeric nomenclature of HPV variants should be used in future epidemiological studies to facilitate a fast and accurate molecular characterization of the HPV16 genome in large-scale studies. In conclusion, with the advent of new molecular techniques such as NGS-WGS, additional studies are warranted to achieve an in-depth and precise characterization of HPV variants in CC and HNSCC, as well as in other HPV–related tumors.

## Figures and Tables

**Table 1:** Alphanumeric and geographical classification systems of the HPV16 variants. The known HPV16 variants, stratified into lineages and sublineages, and the respective GenBank numbers are listed in the table and indicated in the text. The HPV16 variants named B3 (KU053915), B4 (KU053914), C2 (HQ644244), C3 (KU053921), C4 (KU053922), and D4 (KU053933) were only classified with the alphanumeric system [5] (https://pave.niaid.nih.gov/explore/variants/variant_genomes) (accessed on 13 March 2024).

Alphanumeric Classification		Geographical Classification	
Lineage	Sublineage and GenBank Number		
A	A1 (NC_001526)	European	E
A	A2 (AF536179)	European	E
A	A3 (HQ644236)	European	E
A	A4 (AF534061)	Asian	E(As)
B	B1 (AF536180)	African-1	Afr1a
B	B2 (HQ644298)	African-1	Afr1b
B	B3 (KU053915)	_	_
B	B4 (KU053914)	_	_
C	C1 (AF472509)	African-2	Afr2a
C	C2 (HQ644244)	_	_
C	C3 (KU053921)	_	_
C	C4 (KU053922)	_	_
D	D1 (HQ644257)	North American-1	(NA)1
D	D2 (AY686579)	Asian American 2	(AA)2
D	D3 (AF402678)	Asian American 1	(AA)1
D	D4 (KU053933)	_	_

**Table 2:** Main findings from studies reporting (**A**) HPV phylogenetic variants distribution in head and neck tumors from 2000 to 2023, including those exploring also (**B**) specific SNPs in E6 and E7 viral oncogenes.

(A)							
Variant Classification System	Reference	Year	Country	Typing Method	HPV16 Gene	HPV16 Lineages and Number (n) of Cases	Cancer Specimen and Total Sample Size Number (n)
Geographical	[27]	2000	USA	Sanger sequencing	E6	European prototype (n = 39) Asian (n = 9) North American (n = 2) African 1 (n = 2)	HNSCC (n = 253)
Geographical	[28]	2007	Italy	Sanger sequencing	E6	European-German (n = 9) African 2 (n = 2) Asian American (n = 1) Unclassified (n = 1)	HNSCC (n = 115)
Geographical	[29]	2008	USA	Sanger sequencing	E6	European (n = 13) Asian (n = 1)	HNSCC (n = 135)
Geographical	[30]	2014	Italy	Sanger sequencing	L1	European (n = 41) African (n = 10)	OPSC (n = 81)
Geographical	[31]	2016	Brazil	Sanger Sequencing	LCR-E6	European (n = 12) Asian American (n = 9)	HNSCC (n = 186)
Geographical	[32]	2018	USA	Sanger Sequencing	E6-E7	European (n = 77) Asian (n = 6) African 1A (n = 1) African 2A (n = 1) African 2B (n = 1) North American (n = 2) Asian American 1 (n = 2) Asian American 2 (n = 2)	OPSCC (n = 226)
Alphanumeric	[24]	2021	Japan	Sanger sequencing	LCR-E6	A4 (n = 12) A1/A2/A3 (n = 8) D (n = 2) A5 (n = 2)	OPSCC (n = 91)
Alphanumeric	[33]	2021	USA	NGS	WG	A1 (n = 112) A2 (n = 63) A3 (n = 3) A4 (n = 14) C (n = 1) D1 (n = 1) D3 (n = 13) D4 (n = 2)	OPC (n = 259)
Alphanumeric	[34]	2022	USA	NGS	WG	A1 (n = 215) A2 (n = 107) A3 (n = 3) A4 (n = 22) B1 (n = 1) C1 (n = 6) D1 (n = 1) D2 (n = 2) D3 (n = 25) D4 (n = 2)	OPSCC (n = 460)
Alphanumeric	[35]	2022	Greece	Sanger sequencing	E6	A3 (n = 34) D1 (n = 6)	HNSCC (n = 40)
Alphanumeric	[36]	2023	Canada	Sanger sequencing	E6	A1 (n = 38) A2 (n = 54) D2-D3 (n = 2)	HNSCC (n = 94)
**(B)**							
**Reference**		**Year**	**Country**	**Typing Method**	**HPV16 Gene**	**E6 and E7 SNPs** **AA Change and** **Number (n) of Cases**	**Cancer** **Specimen and Total Sample Size Number (n)**
[37]		2004	Germany	Sanger sequencing	E6-E7	350T (n = 6) T350G (L83V) (n = 8) A131G (R10G)/C712A (H51N) (n = 7)	HNSCC (n = 24)
[29]		2008	USA	Sanger sequencing	E6	E-350T (n = 6) E-350G (L83V) (n = 4) E-T131G (R10G) (n = 2)	HNSCC (n = 135)
[38]		2009	Italy	Sanger sequencing	E6	T350G (L83V) (n = 5)	UADT (n = 77)
[39]		2012	Sweden	Sanger sequencing	E6	E-A131G (R10G) (n = 21) E-T350G (L83V) (n = 43)	TSCC (n = 108)
[40]		2015	Japan	Sanger sequencing	E6	E-350T (n = 2) E-350G (L83V) (n = 8)	TSCC (n = 24)
[32]		2018	USA	Sanger sequencing	E6-E7	7392G (L90V) (n = 12) 7173G (R17G) (n = 4) 7754A (H51N) (n = 2)	OPSCC (n = 226)
[35]		2022	Greece	Sanger sequencing	E6	T350G (L83V) (n = 33)	HNSCC (n = 40)
[36]		2023	Canada	Sanger sequencing	E6	350T (n = 33) 350G (L83V) (n = 40)	HNSCC (n = 94)

**HNSCC**: head and neck squamous cell carcinoma; **UADT**: upper aerodigestive tract; **OPC**: oropharyngeal cancer; **OPSCC**: oropharyngeal squamous cell carcinoma; **TSCC**: tonsillar squamous cell carcinoma.; **WG**: whole genome; **NGS**: next generation sequening.

## Data Availability

Data are available upon reasonable request.
